# SLC27A5 Inhibits HBV Replication Through Regulating FXR/SREBP1C/FASN/ACC1 Pathway in HepAD38 Cells

**DOI:** 10.3390/microorganisms14102293

**Published:** 2026-10-09

**Authors:** Mengren Cun, Yongxin Liu, Xinrong Ma, Xinran Zhang, Yue Feng, Xueshan Xia, A-Mei Zhang

**Affiliations:** Faculty of Life Science and Technology & Yunnan Province Key Laboratory of Public Health and Biosafety, Kunming University of Science and Technology, Kunming 650500, China; mengren1999@163.com (M.C.); xinran122130@163.com (X.Z.);

**Keywords:** SLC27A5, inhibit HBV replication, FXR/SREBP1C/FASN/ACC1, lipid metabolism

## Abstract

Hepatitis B virus (HBV) infection can lead to hepatitis B, which may eventually progress to cirrhosis or hepatocellular carcinoma (HCC). Host lipid metabolism plays a critical role in HBV replication, and HBV infection also impairs lipid homeostasis. The solute carrier family 27 member 5 (*SLC27A5*) gene, which encodes a long-chain fatty acid transporter and bile acid–CoA ligase, has been reported to be associated with cirrhosis and HCC. However, whether SLC27A5 influences HBV infection or replication remains unclear. In this study, the *SLC27A5* gene was overexpressed or silenced in HepAD38 cells. Results show that SLC27A5 overexpressing suppresses HBV replication. Given the primary role of SLC27A5 in bile acid metabolism, we hypothesized that SLC27A5 might inhibit HBV replication via regulating the FXR/SREBP1C/FASN/ACC1 signaling pathway. Subsequent experiments confirmed that overexpression of SLC27A5 upregulated FXR expression levels and downregulated mRNA levels of the *SREBP1C*, *FASN*, and *ACC1* genes. Consequently, lipid synthesis was suppressed in SLC27A5-overexpressing cells, with significantly reduced levels of lipid droplets and triglycerides. Furthermore, SLC27A5 might collaborate with Entecavir to inhibit HBV replication. In summary, we demonstrated that SLC27A5 inhibited HBV replication through regulating FXR/SREBP1C/FASN/ACC1 and decreasing lipid synthesis.

## 1. Introduction

Hepatitis B virus (HBV) infection remains a major global public health burden and a leading cause of hepatocellular carcinoma (HCC). Chronic HBV carriers have a significantly elevated risk of developing HCC, and approximately 84% of HCC cases in China are attributable to HBV infection [1,2]. In 2022, the World Health Organization (WHO) reported 254 million cases of chronic HBV infection worldwide, resulting in approximately 1.1 million annual deaths. Despite the severity of this disease, diagnosis and treatment coverage remain alarmingly low, at only 13.4% and 2.6%, respectively [3,4]. Furthermore, although vaccination programs have been implemented, birth-dose immunization coverage remains inadequate, leaving individuals susceptible to HBV infection in adulthood [5].

HBV entry, replication, and pathogenesis depend heavily on host lipid metabolism. The viral envelope is rich in lipids, and HBV infection can upregulate cholesterol biosynthesis to facilitate its own replication [6]. HBV infection also induces lipid metabolism disorders in hepatocytes, including aberrant lipid accumulation and fatty acid dysregulation, which further contribute to chronic liver injury and hepatic steatosis [7]. The HBV regulatory protein HBx can activate the expression of genes involved in host lipogenic signaling pathways, thereby promoting HBV genome transcription and viral replication. Moreover, HBx impairs host immune responses and lipid metabolism by disrupting mitochondrial function, leading to increased production of reactive oxygen species (ROS) and excessive lipid peroxidation [7]. These pathological alterations promote intracellular lipid droplet accumulation and further change the hepatocyte environment, which is closely correlated with both HBV replication and disease progression [8]. Zou et al. queried 18 datasets and screened differentially expressed proteins between tumorous and adjacent nontumorous tissues of HBV-related HCC patients. They suggested that the lipid-metabolism-related gene ANGPTL6 could be used as a biomarker for the early diagnosis of HBV-related HCC [9]. Given the pivotal role of lipid metabolism in HBV infection, targeting host lipid metabolism represents a promising therapeutic strategy against HBV infection [9,10].

The solute carrier family 27 member 5 (*SLC27A5*) gene encodes fatty acid transport protein-5 (FATP5), a liver-specific protein that primarily functions as a long-chain fatty acid transporter and a bile acid–CoA ligase. It has emerged in recent years as a critical modulator of hepatic disease progression that extends far beyond canonical metabolic regulation. SLC27A5 suppresses TXNRD1 expression via the KEAP1/NRF2 pathway in HCC patients. Thus, combination therapy of sorafenib and NRF2/TXNRD1 inhibitors may be a promising strategy in personalized HCC treatment [11]. SLC27A5 is also reported to regulate HCC progression in a non-canonical way. SLC27A5 can interact with IGF2BP3 to modulate PIP4K2A pre-mRNA splicing. Conversely, SLC27A5 over-expression exerts an inhibitory effect on HCC metastasis via reducing expression of the PIP4K2A-S isoform [12]. Thus, the downregulated expression of *SLC27A5* in HCC patient tissues suggests that it may exert a potential tumor-suppressive effect [11,12]. Wu et al. reported that SLC27A5 is transcriptionally downregulated by the transcriptional repressor RUNX family transcription factor 2 (RUNX2), and loss of SLC27A5 also promotes liver fibrosis via activation of hepatic stellate cells (HSCs) [13]. Collectively, these studies indicate that SLC27A5 plays important roles in hepatic pathogenesis through diverse regulatory mechanisms.

HBV is recognized as a “metabolic virus” and one of the leading causes of HCC. Its infection, replication, and pathogenic processes are all closely related to host lipid metabolism. Despite the non-negligible role of SLC27A5 in the pathogenesis of HCC, the molecular interplay between HBV and SLC27A5-mediated lipid metabolic reprogramming remains largely under-explored. Existing studies have not systematically defined whether SLC27A5 alteration directly modulates HBV replication, viral gene expression, or the progression from chronic HBV infection to fibrosis and HCC. It is necessary to investigate whether SLC27A5 modulates HBV replication, and its anti-HBV mechanism needs to be reveal.

In this study, we found that SLC27A5 decreased lipid droplets through regulating the FXR/SREBP1C/FASN/ACC1 pathway and inhibited HBV replication in HepAD38 cells.

## 2. Materials and Methods

### 2.1. Cell Culture, Plasmids Construction, and Transfection

The human hepatoma cell line HepAD38, which carries 1.3-fold copies of the HBV genome and stably secretes infectious HBV particles, was obtained from the Cell Bank of the Chinese Academy of Sciences (Shanghai, China) and continuously maintained in our laboratory. Cells were cultured in high-glucose Dulbecco’s modified Eagle’s medium (Procell, Wuhan, China) supplemented with 10% fetal bovine serum (Sigma-Aldrich, Darmstadt, Germany), 100 U/mL penicillin, and 100 μg/mL streptomycin (Procell, China). All cells were incubated at 37 °C in a humidified incubator with 5% CO_2_.

The full-length coding sequence (CDS) of the *SLC27A5* gene was amplified using the primer pair SLC27A5-F (5′-G*GAATTC*ATGGGTGTCAGGCAACAGTTG-3′) and SLC27A5-R (5′-GG*GGTACC*GAGCCTCCAGGTTCCCTCACA-3′). Restriction endonuclease sites—an *Eco*RI site in SLC27A5-F and a *Kpn*I site in SLC27A5-R—were introduced at the 5′ ends of the respective primers (sequences underlined). The PCR product was cloned into the pCMV-C-Flag plasmid. To generate SLC27A5-knockout cell lines, a single-guide RNA sequence (5′-CACCGCATCTACAAAGGTGTCAGG-3′) was cloned into the CRISPR/Cas9 lentiviral vector lentiCRISPR v2. Lentivirus particles were produced by co-transfecting HEK-293T cells with plasmids lentiCRISPR v2, pMD2.G, and psPAX2.

All constructed plasmids were transfected into HepAD38 cells using Lipofectamine 3000 reagent (Invitrogen, Carlsbad, CA, USA). Entecavir (ETV; Meilunbio, Dalian, China) was used as the positive anti-HBV agent at a final concentration of 20 μM.

### 2.2. HepAD38 Cells with Knockout of the SLC27A5 Gene

A lentivirus-mediated CRISPR/Cas9 gene-editing system was used to generate SLC27A5-knockout HepAD38 cell lines. A single-guide RNA (sgRNA) targeting the 3′-UTR of the *SLC27A5* gene (sequence: 5′-CACCGCATCTACAAAGGTGTCAGG-3′) was cloned into the pLentiCRISPRv2 vector. The recombinant pLentiCRISPRv2—SLC27A5-KO plasmid, together with the envelope plasmid pMD2.G and the packaging plasmid psPAX2, was co-transfected into HEK-293T cells at a mass ratio of 10:4:2. Viral supernatants were harvested at 72 h post-transfection. HepAD38 cells were then transduced with the concentrated lentivirus, followed by puromycin selection at a final concentration of 2 μg/mL to establish a monoclonal SLC27A5-knockout stable HepAD38 cell line.

### 2.3. Detection of HBV Viral Loads, HBsAg, and HBeAg Levels

Total RNA was extracted and reverse-transcribed into cDNA using the Evo M-MLV Plus First Strand cDNA Synthesis Kit (Accurate Biology, Changsha, China). Relative mRNA expression levels were quantified via quantitative real-time PCR (qRT-PCR) using ChamQ SYBR qPCR Master Mix (Vazyme, Nanjing, China) at 48 h post-transfection. The housekeeping gene *GAPDH* was selected as the internal reference for normalization, and relative gene expression was calculated using the 2^−ΔΔCt^ method across three independent biological replicates. The primer sequences used are listed in Table S1.

At 48 h post-transfection, cell culture supernatants were collected and centrifuged at 12,000× *g* for 5 min at 4 °C to remove floating cells and insoluble debris. HBV DNA was extracted using a viral DNA/RNA extraction kit (Tiangen, Beijing, China) following the manufacturer’s instructions. Then, HBV viral loads were quantified by qPRC using a standardized HBV DNA quantitative detection kit, with a 10-fold serially diluted HBV DNA standard curve. Secreted HBsAg and HBeAg levels in the same harvested cell culture supernatants were measured using HBsAg/HBeAg ELISA kits (Kehua Bio-engineering, Shanghai, China) strictly in accordance with the manufacturer’s instructions. In brief, 50 μL of diluted supernatant sample was added to each pre-coated microwell, alongside calibrated standard samples and negative/quality control samples provided with the kit. After 60 min of incubation at 37 °C, plates were washed five times to remove unbound components, followed by the addition of detection antibody and another 30 min incubation at 37 °C. After a second round of thorough washing, chromogenic substrates A and B were added sequentially, and the reaction was terminated with stop solution after 15 min of color development in the dark. The absorbance value was immediately measured using a microplate reader at 450 nm, and the final antigen concentrations were calculated based on the standard curve, with all data presented as the relative fold change normalized to the vector control group from three independent replicate experiments.

### 2.4. Western Blot

Total proteins were extracted and quantified using BCA Protein Assay Kit (Beyotime, Shanghai, China). Subsequently, equal amounts of denatured proteins were separated via SDS-PAGE and transferred onto PVDF membranes for Western blot immunoblotting analysis. The following antibodies were used to detect target protein expression levels across different treatment groups: anti-SLC27A5 (1:1000, Invitrogen), anti-NRF2 (1:1000, ABclonal, Wuhan, China), anti-FXR (1:500, ABclonal), anti-ubiquitin (1:1000, Proteintech, Rosemont, IL, USA), anti-GAPDH (1:10,000, Proteintech), goat anti-rabbit IgG H&L (HRP) (1:5000, Abcam, Cambridge, Britain), and goat anti-mouse IgG H&L (HRP) (1:5000, Abcam).

### 2.5. Measurement of Total Bile Acid, Triglyceride, and Lipid Droplets

Total intracellular bile acid and triglyceride levels were measured using the total bile acid content assay kits (CAT: BC6305) and the triglyceride content assay kits (CAT: BC0625), following the manufacturer’s instruction manual (Solarbio, Beijing, China). In brief, the treated HepAD38 cells were harvested, resuspended in extraction buffer, and lysed by sonication, followed by centrifugation for subsequent biochemical detection. Finally, the processed samples and corresponding standard substances were mixed with reagents, and the absorbance was measured at 405 nm and 420 nm for total bile acid and triglyceride detection, respectively. Cellular lipid droplets were visualized with the Lipi-Deep Red small-molecule fluorescent probe (Dojindo, kumamoto, Japan). Briefly, cells were incubated with the probe at a final concentration of 0.1 µM for 30 min at 37 °C, and subsequently observed under a laser scanning confocal microscope (Nikon, Tokyo Metropolis, Japan) with a designated excitation wavelength of 640 nm.

### 2.6. Statistical Analysis

All data are presented as the mean ± standard deviation (SD) from at least three independent biological replicates. All in vitro experiments were performed in technical triplicate. Statistical analyses were performed using an unpaired Student’s *t*-test or one-way ANOVA, as appropriate, with GraphPad Prism 9.0. A *p*-value of less than 0.05 was considered statistically significant.

## 3. Results

### 3.1. SLC27A5 Suppressing HBV Replication

**Figure 1:**
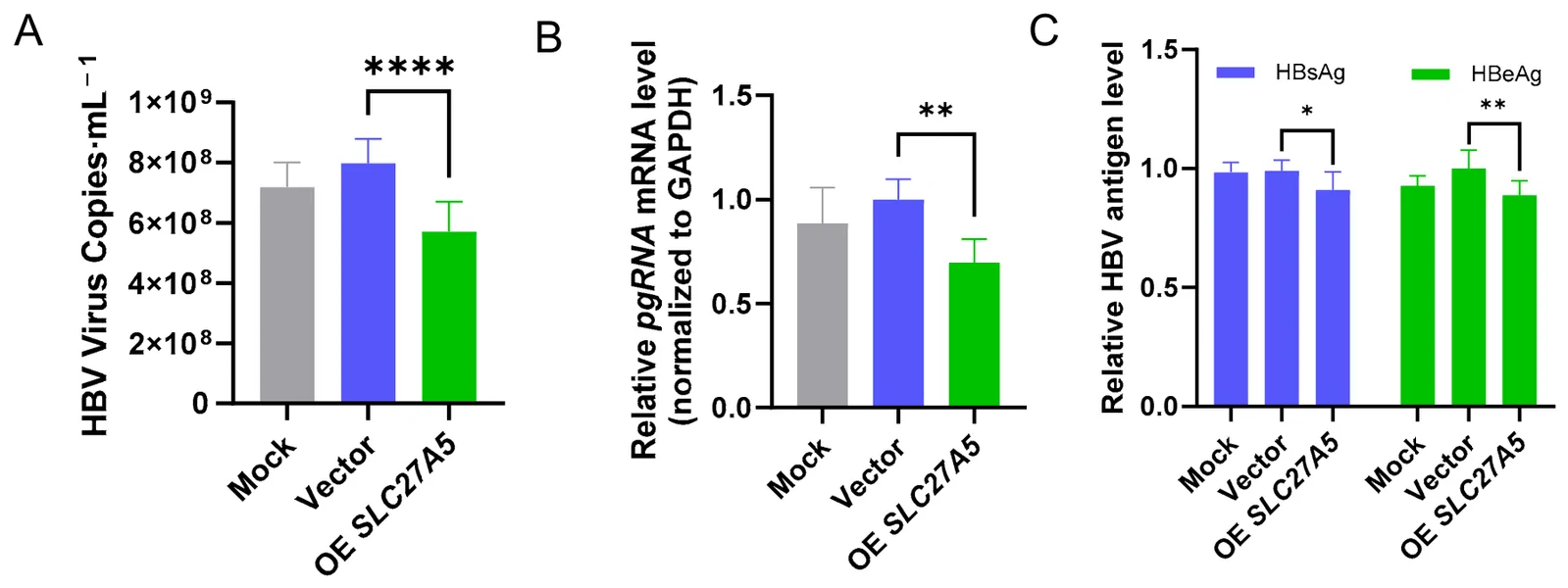
**Overexpression of SLC27A5 suppresses HBV replication.** HepAD38 cells transfected with *SLC27A5* decrease HBV DNA copies (**A**), pgRNA levels (**B**), and HBsAg and HBeAg (**C**). * *p* < 0.05, ** *p* < 0.01, **** *p* < 0.0001.

To investigate whether SLC27A5 affects HBV replication, HBV DNA copies were measured in HepAD38 cells with or without SLC27A5 overexpression. HBV DNA levels were significantly reduced in SLC27A5-overexpressing cells (Figure 1A). In addition, overexpression of SLC27A5 markedly downregulated the levels of HBV pre-genomic RNA (pgRNA), secreted HBsAg and HBeAg in cell culture supernatants (Figure 1B,C). Taken together, these results suggest that overexpression of the *SLC27A5* gene significantly inhibits both HBV replication and virion release.

### 3.2. SLC27A5 Regulated FXR/SREBP1C/FASN/ACC1 Signaling Pathway via Bile Acid

**Figure 2:**
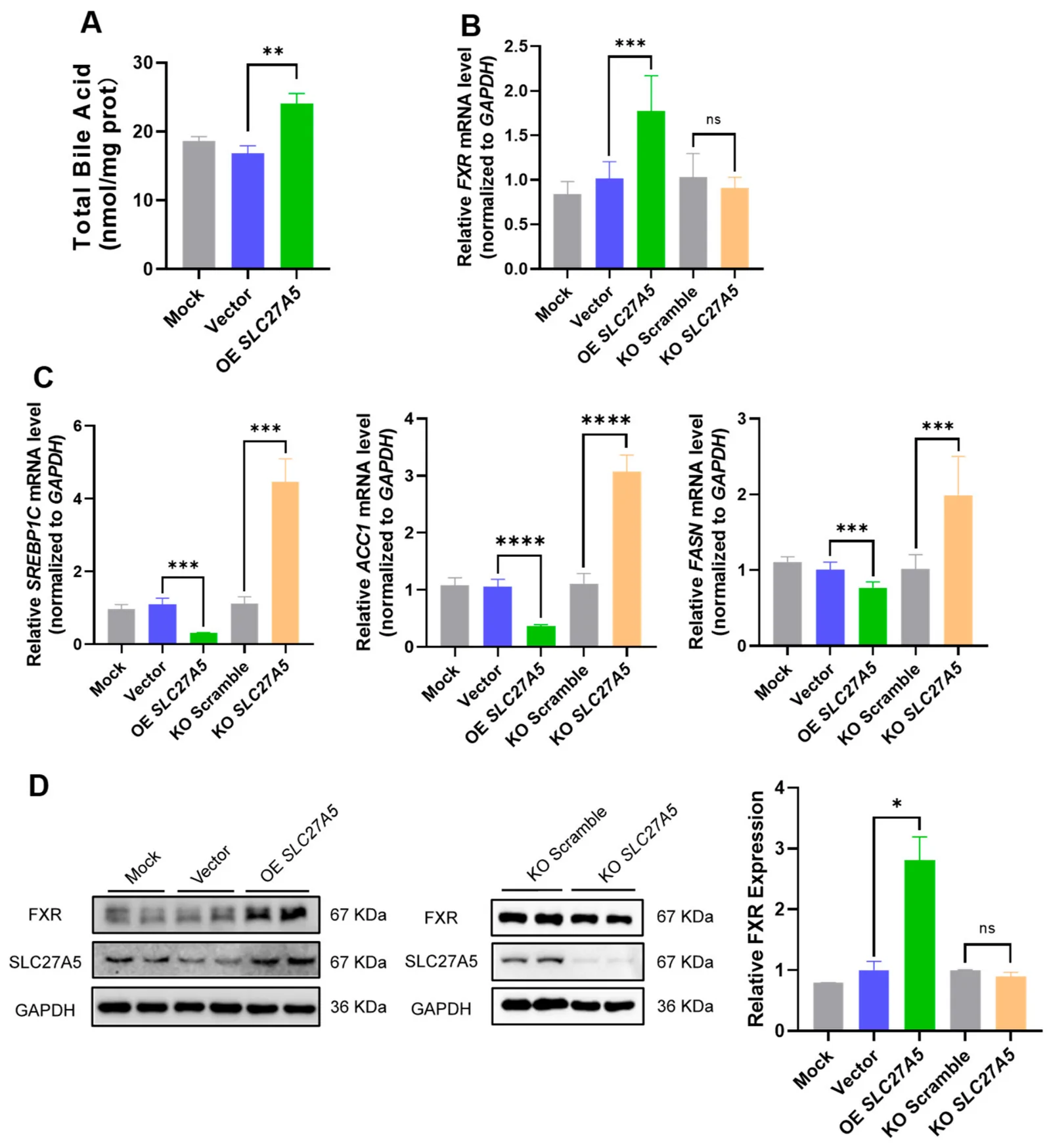
**SLC27A5 modulates FXR/SREBP1C/FASN/ACC1 pathway.** (**A**) Overexpression of SLC27A5 increased total bile acid (TBA) levels. (**B**) Overexpression of SLC27A5 significantly upregulated mRNA level of FXR. (**C**) Overexpression of SLC27A5 significantly decreased mRNA level of SREBP1c, FASN, and ACC1. (**D**) Overexpression of SLC27A5 increased FXR protein expression level. * *p* < 0.05, ** *p* < 0.01, *** *p* < 0.001, **** *p* < 0.0001; ns, not significant.

A main biochemical function of SLC27A5 is to mediate the intracellular binding and trafficking of bile acids, which are natural ligands of the farnesoid X receptor (FXR). We hypothesize that SLC27A5 suppresses HBV replication via regulating FXR expression. First, total bile acid levels were significantly increased in SLC27A5-overexpressing cells compared with control cells (Figure 2A), and this SLC27A5-dependent bile acid accumulation led to marked upregulation of FXR mRNA levels (Figure 2B). Subsequently, activated FXR further suppressed the mRNA expression levels of the *SREBP1C* gene, and consequently reduced the mRNA levels of its downstream target genes *ACC1* and *FASN* (Figure 2C). In contrast, SLC27A5 knockout resulted in the opposite phenotypic changes (Figure 2B,C). Immunoblotting analysis confirmed that FXR protein levels were increased in SLC27A5-overexpressing cells, while they were substantially reduced in SLC27A5-knockout cells (Figure 2D). These results indicate that SLC27A5 regulates the FXR/SREBP1C/FASN/ACC1 signaling pathway by modulating bile acid homeostasis.

### 3.3. SLC27A5 Overexpression Inhibited Lipid Synthesis

**Figure 3:**
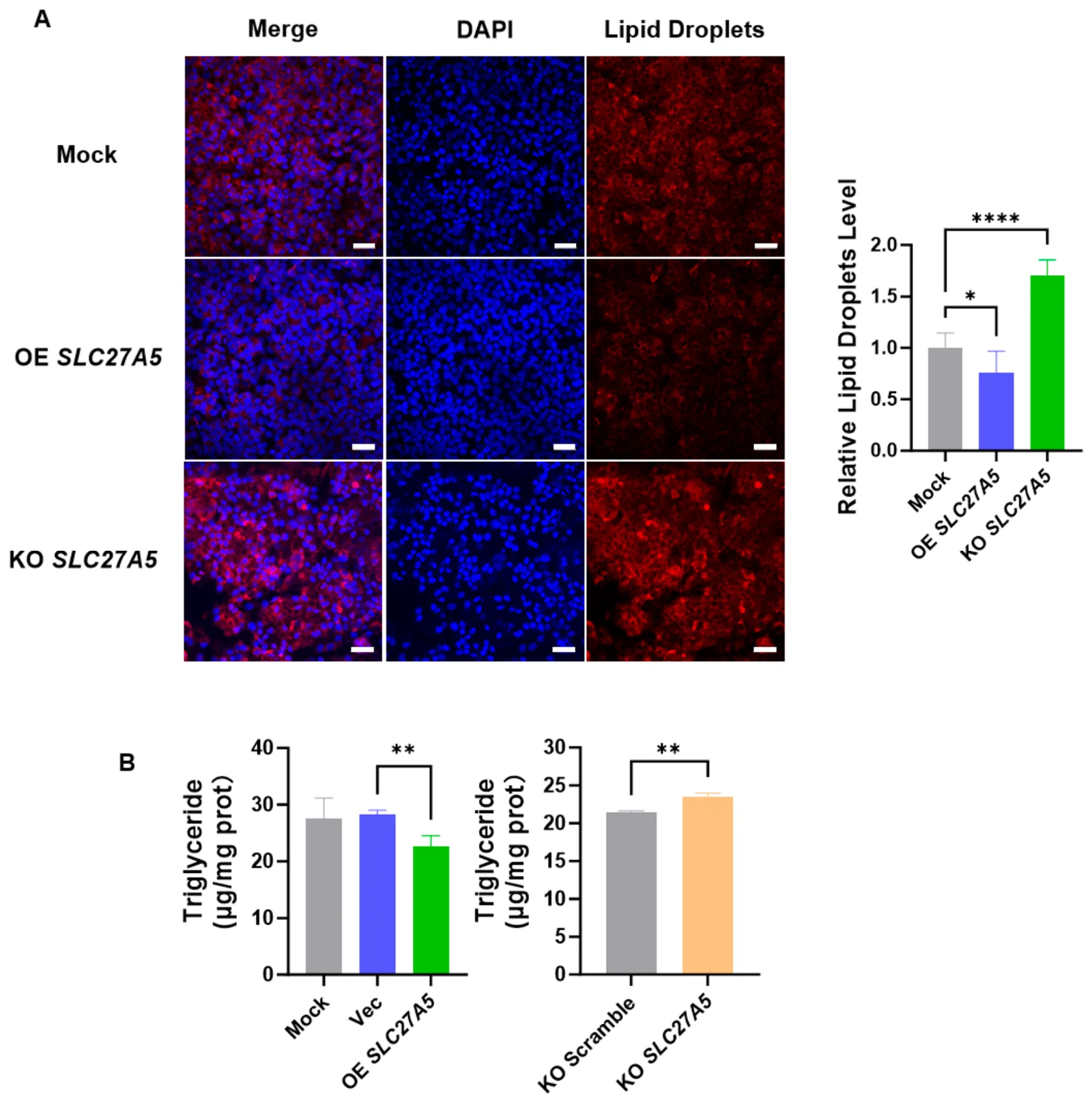
**SLC27A5 downregulates host lipid synthesis.** (**A**) Number of lipid droplets significantly decreased in cells overexpressing SLC27A5. Blue indicates the nuclei stained with DAPI; red represents the number of intracellular lipid droplets. The scale bar is 10 × 10. (**B**) Triglyceride (TG) levels significantly decreased in cells overexpressing SLC27A5. * *p* < 0.05, ** *p* < 0.01, **** *p* < 0.0001.

To investigate whether SLC27A5 overexpression suppressed lipogenesis via the SREBP1C/FASN/ACC1 signaling pathway, we examined neutral lipid droplets and intracellular triglyceride (TG) content. The number of neutral lipid droplets was significantly reduced in SLC27A5-overexpressing cells, while it was markedly increased in SLC27A5-knockout cells (Figure 3A). Consistent with the changes in lipid droplet levels, TG levels were also significantly decreased under SLC27A5 overexpression, and markedly elevated in SLC27A5-knockout cells (Figure 3B). Collectively, these results indicate that SLC27A5 overexpression attenuates lipogenesis by suppressing the SREBP1C/FASN/ACC1 signaling pathway.

### 3.4. SLC27A5 Potentiates the Anti-HBV Activity of Entecavir

**Figure 4:**
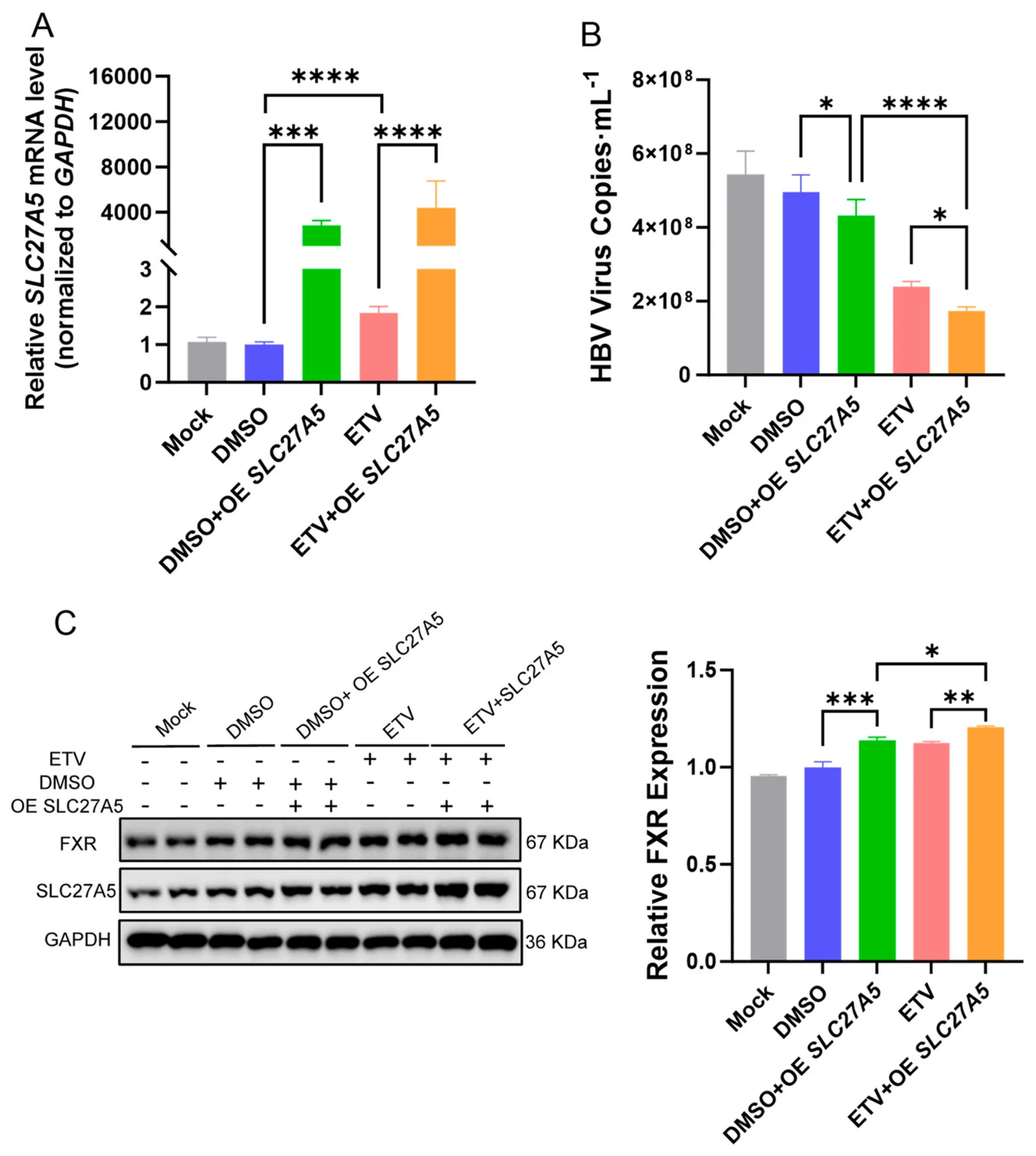
**SLC27A5 plays a synergistic role with ETV in inhibiting HBV replication.** (**A**) Expression level of SLC27A5 increased in cells treated with 20 μM ETV. (**B**) HBV viral loads markedly reduced in cells treated with ETV and transfected with SLC27A5. (**C**) FXR expression levels were significantly increased in cells treated with ETV and transfected with SLC27A5. * *p* < 0.05, ** *p* < 0.01, *** *p* < 0.001, **** *p* < 0.0001.

FXR plays a pivotal role in HBV DNA transcription, and its pharmacological activation is known to robustly suppress total HBV RNA synthesis. We therefore hypothesize that SLC27A5 might collaborate with Entecavir (ETV) to inhibit HBV replication. To test this hypothesis, we measured SLC27A5 mRNA expression across five distinct experimental groups: control, DMSO-treated, DMSO-treated with SLC27A5 overexpression, ETV-treated, and ETV treated with SLC27A5 overexpression. ETV treatment significantly upregulated SLC27A5 mRNA levels (Figure 4A). Consistent with this observation, HBV viral loads were significantly reduced in cells with ETV and SLC27A5 overexpression (Figure 4B). Furthermore, FXR expression was enhanced by either SLC27A5 overexpression or ETV monotherapy. Notably, the combined treatment of ETV and SLC27A5 induced a significant upregulation of FXR levels compared to the effect of each single treatment (Figure 4C). These findings suggest that SLC27A5 might collaborate with ETV to inhibit HBV replication. This potential adjuvant effect of SLC27A5 is most likely mediated by the sustained upregulation of FXR signaling.

## 4. Discussion

**Figure 5:**
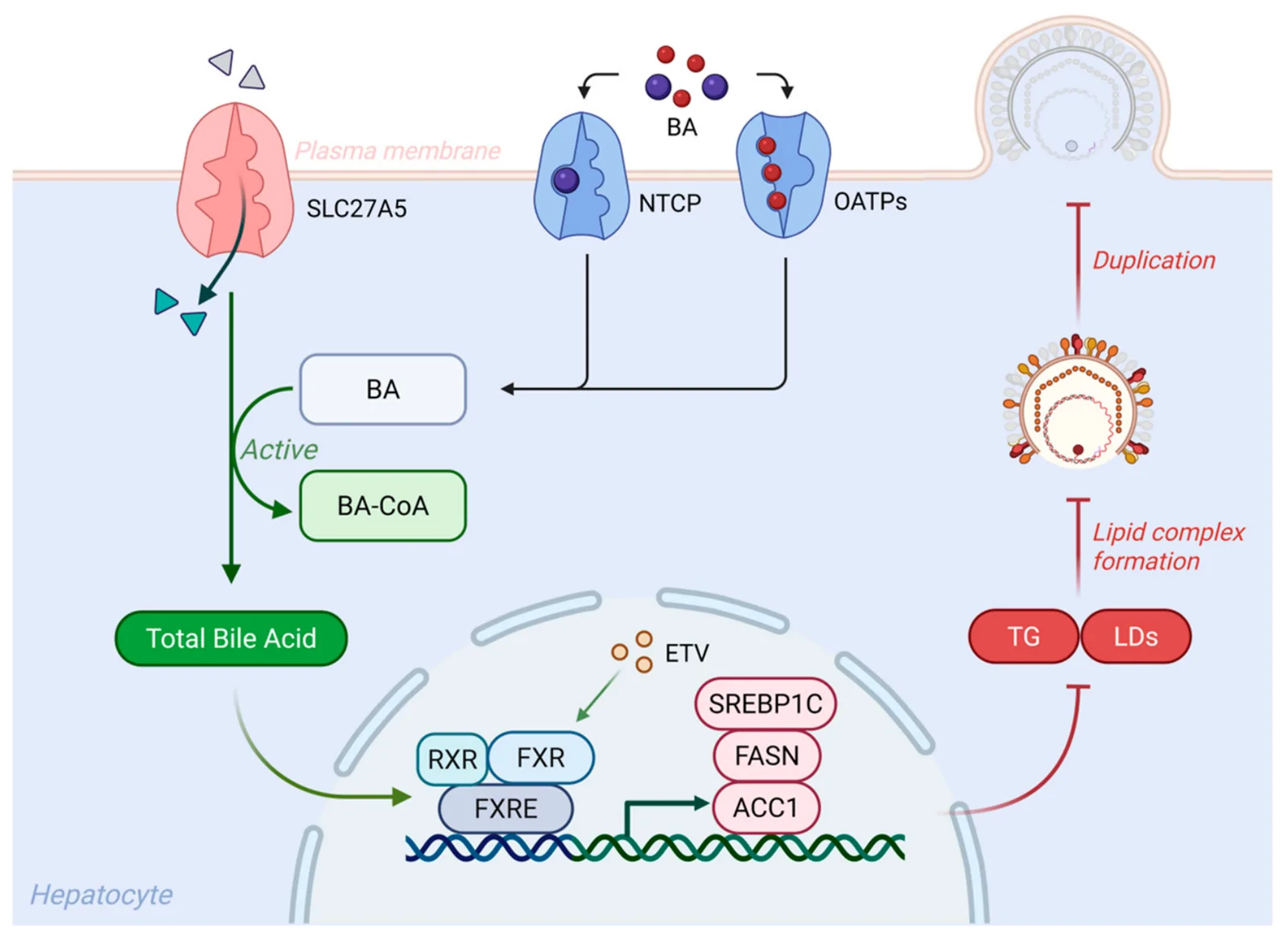
**The mechanistic model of SLC27A5 inhibiting HBV replication**. SLC27A5 inhibits HBV replication through regulating FXR/SREBP1C/FASN/ACC1 pathway.

HBV is an enveloped DNA virus that is highly dependent on host lipids to support its viral replication, nucleocapsid assembly, and envelope formation. Accumulating evidence demonstrates that HBV actively reprograms host lipid metabolism to generate membrane structures and lipid microenvironment for robust viral replication [14]. Beyond their conventional roles as structural components, lipids modulate membrane fluidity, lipid droplet dynamics, and lipid-dependent signaling pathways tightly linked to the HBV life cycle. Consequently, the host lipid metabolic state is considered a critical determinant of HBV replication efficiency. In this study, overexpression of SLC27A5 led to a marked reduction in triglyceride content and diminished lipid droplet accumulation, alongside decreased levels of HBV DNA, pgRNA, and HBsAg. These observations suggest that SLC27A5-mediated lipid metabolic remodeling contributes to the host antiviral effect against HBV (Figure 5).

SLC27A5 is a liver-enriched fatty acid transporter that has been characterized as a hepatic bile acid–CoA ligase. It facilitates the conjugation of free bile acids to bile acid–CoA intermediates, thereby shaping the intracellular bile acid pool and regulating total bile acid abundance [15,16]. In metabolic disorders such as non-alcoholic fatty liver disease (NAFLD), SLC27A5 deficiency drives the abnormal accumulation of free bile acids, aberrant activation of stress-responsive transcription factors, and progressive exacerbation of liver injury. In contrast, its physiological expression supports metabolic homeostasis and exerts prominent hepatoprotective effects [13,17]. Although direct evidence linking SLC27A5 to viral infection remains limited, host lipid metabolic regulators have been implicated in the replication of multiple viruses [18,19]. This conserved regulatory pattern suggests that SLC27A5 may indirectly modulate HBV replication through its intrinsic hepatic metabolic regulatory functions. Consistently, our findings demonstrate that SLC27A5 upregulation is associated with improved lipid metabolism and enhanced anti-HBV activity, further establishing its previously unrecognized role as a key host metabolic restriction factor for HBV.

Nucleotide analogs can influence hepatic lipid homeostasis. Given that bile acids act as endogenous ligands for FXR, their composition and concentration directly influence FXR activation [20]. In this study, SLC27A5 overexpression elevated intracellular total bile acid levels and concomitantly upregulated FXR expression at both the mRNA and protein levels, indicating enhanced FXR signaling through the regulation of bile acid homeostasis. Previous studies have demonstrated that FXR activation suppresses SREBP1C transcriptional activity and downregulates genes involved in fatty acid synthesis, thereby inhibiting hepatic lipogenesis [21]. Furthermore, FXR activation has been reported to directly or indirectly suppress HBV transcription and replication, highlighting its role in regulating the viral life cycle [22]. Collectively, these findings suggest that SLC27A5 may inhibit HBV replication by modulating the bile acid–FXR axis, which in turn suppresses the SREBP1C/FASN/ACC1 signaling pathway and reduces the lipid availability essential for viral replication.

Although FXR activation typically triggers the SHP-CYP7A1 negative feedback loop, this regulatory process is temporally dynamic [23]. At the early stage, increased bile acid conjugation and intracellular retention may occur prior to the onset of feedback inhibition, thereby providing a transient window for FXR activation [24]. Moreover, distinct bile acid species exhibit differential FXR agonistic activities, and shifts in bile acid composition may favor FXR activation even under the condition of elevated total bile acid levels [25]. These mechanisms may explain the concurrent elevation of total bile acids and FXR activation observed in this study.

With respect to antiviral therapy, ETV treatment not only suppressed HBV replication but also upregulated the protein expression of both SLC27A5 and FXR. Although ETV primarily acts as an inhibitor of HBV polymerase, accumulating evidence demonstrates that nucleotide analogs can reshape host metabolic networks during the process of viral suppression [26]. Metabolomic studies further revealed that ETV alleviated HBV-induced metabolic stress and inflammation, accompanied by widespread alterations in bile acid and lipid profiles [27]. In this context, ETV can modulate the bile acid–FXR axis via metabolic reprogramming, thereby regulating the expression of metabolism-related genes, including the *SLC27A5* gene [28]. In line with these findings, our results show that ETV induces SLC27A5 expression, and that SLC27A5 overexpression might collaborate with ETV to inhibit HBV replication, implying a potential cooperative interaction between antiviral therapy and host lipid metabolism.

Collectively, these findings suggest that SLC27A5 enhances bile acid conjugation and intracellular retention, thereby generating a bile acid pool that promotes ligand-dependent FXR activation. By coordinating bile acid and lipid metabolism, SLC27A5 establishes a cellular state that suppresses HBV replication. This metabolic mechanism plausibly explains the antiviral effects mediated by SLC27A5 upregulation, and highlights the SLC27A5-FXR axis as a potential host-directed therapeutic target for HBV infection.

Although our present study identifies a significant inhibitory effect of SLC27A5 on HBV replication and its potential suppressive mechanism, several limitations and unresolved concerns remain to be addressed before the findings can be used as the theoretical basis for HBV treatment in the clinic. First, the current data do not confirm whether the observed antiviral effects and host lipid metabolism alterations can recapitulate the complete HBV life cycle, as the HepAD38 cell line does not fully mirror the physiological complexity of HBV infection in human hepatocytes. The present results should be validated in other cell models, such as NTCP-expressing HepG2 cells. Second, while the study proposes a potential association between SLC27A5 and FXR, the causal relationship between these two genes needs to be definitively established. There is a lack of evidence from both pharmacological and genetic loss-of-function studies targeting FXR to verify that inhibition of this nuclear receptor can fully reverse the inhibitory effects induced by SLC27A. In addition, complementary FXR activation or rescue assays in SLC27A5-knockout cellular models are further needed. These unresolved problems highlight that additional independent validation in more physiologically relevant HBV infection systems, i.e., HBV infection in NTCP-expressing human hepatocytes, is required to rigorously confirm this study.

## 5. Conclusions

SLC27A5 modulates intracellular bile acid homeostasis, triggering the activation of the FXR signaling pathway and the remodeling of hepatic lipid metabolism. These rearrangements disrupt the assembly of HBV-associated lipid complexes, thereby suppressing viral replication and virion spread.

## Data Availability

The original contributions presented in this study are included in the article/Supplementary Materials. Further inquiries can be directed to the corresponding author.

## Supplementary Materials

The following supporting information can be downloaded at: https://www.mdpi.com/article/10.3390/microorganisms14102293/s1, Table S1: Specific primers for RT-qPCR.
