# Supplementary material for: SLC27A5 Inhibits HBV Replication Through Regulating FXR/SREBP1C/FASN/ACC1 Pathway in HepAD38 Cells
Source: Microorganisms. 2026 Oct 9;14(10):2293. doi: 10.3390/microorganisms14102293 (PMC13707536; doi:10.3390/microorganisms14102293)
Supplement: Supplementary file 1 [file microorganisms-14-02293-s001.zip › microorganisms-4565311-supplementary.pdf]

Table S1 Specific primers for RT-qPCR

Target	Forward Primer (5'- 3')	Reverse Primer (5'- 3')
SLC27A5	CTGCCTCCTCCGAATGCT	TTGCTGGCTTACCACCTTG
GAPDH	CAGGAGGCATTGCTGATGAT	GAAGGCTGGGGCTCATTT
HBV DNA	GTTGCCCGTTTGTCCTCTAATTC	GGAGGGATACATAGAGGTTCCTT
pgRNA	CTCAATCTCGGGAATCTCAATGT	TGGATAAAACCTAGCAGGCATAAT
FXR	CGTCAGCAGGGAGGATCAAA	CCTTTACACCCCTCACAGGTC
FASN	TGTACGCCACCATCCTGAAC	TGGTACAACGAGCGGATGAG
SREBP1C	CTGACACGCTTCTTCCTGAG	CCCATCCACGAAGAAACGG
